# Synthesis, Characterization, and Photocatalytic Evaluation of Manganese (III) Phthalocyanine Sensitized ZnWO_4_ (ZnWO_4_MnPc) for Bisphenol A Degradation under UV Irradiation

**DOI:** 10.3390/nano10112139

**Published:** 2020-10-27

**Authors:** Chukwuka Bethel Anucha, Ilknur Altin, Zekeriya Biyiklioglu, Emin Bacaksiz, Ismail Polat, Vassilis N. Stathopoulos

**Affiliations:** 1 Department of Chemistry, Faculty of Science, Karadeniz Technical University, 61080 Trabzon, Turkey; ilknurtatlidil@ktu.edu.tr (I.A.); zekeriyab@ktu.edu.tr (Z.B.); 2 Department of Physics, Faculty of Science, Karadeniz Technical University, 61080 Trabzon, Turkey; eminb@ktu.edu.tr; 3 Department of Energy Systems, Faculty of Technology, Karadeniz Technical University, 61080 Trabzon, Turkey; ipolat@ktu.edu.tr; 4 Laboratory of Chemistry and Materials Technology, General (Core) Department, National and Kapodistrian University of Athens, Psachna Campus, 34400 Evia, Greece; vasta@uoa.gr

**Keywords:** photocatalysis, hydrothermal autoclave, ZnWO_4_, manganese (III) phthalocyanine sensitized ZnWO_4_ photocatalyst, emerging contaminants, bisphenol A

## Abstract

ZnWO_4_MnPc was synthesized via a hydrothermal autoclave method with 1 wt.% manganese (iii) phthalocyanine content. The material was characterized for its structural and morphological features via X-ray diffraction (XRD) spectroscopy, Fourier transform infrared (FTIR) spectroscopy, transmission emission microscopy (TEM), scanning electron microscopy-Energy dispersive X-ray spectroscopy (SEM-EDX), N_2_ adsorption–desorption at 77K, X-ray photoelectron spectroscopy (XPS), and UV-visible/diffuse reflectance spectroscopy(UV-vis/DRS). ZnWO_4_MnPc photocatalytic performance was tested on the degradation of bisphenol A (BPA). The ZnWO_4_MnPc material removed 60% of BPA after 4 h of 365 nm UV irradiation. Degradation process improved significantly to about 80% removal in the presence of added 5 mM H_2_O_2_ after 4 h irradiation. Almost 100% removal was achieved after 30 min under 450 nm visible light irradiation in the presence of same concentration of H_2_O_2_. The effect of ions and humic acid (HA) towards BPA removal was also investigated.

## 1. Introduction

Economic advancement and improved quality of life at the turn of the 20th century has come with a price as many produced and spent synthetic molecules make their way into the environment posing a risk to life in water and on land [1,2]. Majority of these compounds being persistent in nature and with a high tendency to bioaccumulate cuts across a wide spectrum of classes with respect to their applications such as pharmaceuticals and personal care products (PPCPs), pesticides, organic solvents, and industrial chemicals [2]. Bisphenol A (2,2-bis (4-hydroxyphenyl) propane, an industrial chemical extensively used as starting material for epoxy and polycarbonate plastics have been identified as endocrine disrupting substance with reported estrogenic and androgenic potencies leading to hormonal malfunctions in various organisms [3,4,5,6,7,8,9,10,11,12]. Bisphenol A (BPA) identified as a contaminant of emerging concern (CEC) by Network of Reference Laboratories, Research Centers and Related Organization for the Monitoring of Emerging Environmental Substances (NORMAN) network [13], elude conventional wastewater treatment plant (WWTP) strategies due to its refractoriness. Therefore, to reduce the human and environmental potential risks associated with end of pipe biorecalcitrants such as the likes of BPA and other related CEC compounds, further improvement of WWTP performance is needed [14]. Amongst several techniques that have been developed to tackle the challenge of detoxification of organic compound contaminated wastewaters, and at least bring down their pollution parameters to a satisfactory level, advanced oxidation process (AOPs) have been the most studied [7]. At the center point of advanced oxidation processes (AOPs) is TiO_2_ semiconductor-based heterogeneous photocatalysis [7]. TiO_2_ (Titania) has played a crucial role in the development of semiconductor photocatalysis over the last decades, thanks to its unmatching characteristic properties of long-term stability, chemical inertness, corrosion resistance, and non-toxicity [15]. The numerous benefits notwithstanding, titania application is still limited to high intensity solar radiation absorption region due to high energy band gap of 3.2 eV [16]. To extend the absorption spectrum range and cultivate more solar radiation energy in the applicability of titania photocatalysis, certain strategies like doping, semiconductor coupling and, dye sensitization have been adopted [16]. Although titanium dioxide (TiO_2_, titania) has been well known and established in semiconductor photocatalysis, other materials aimed at band gap reduction and overall performance improvement have equally being sought and investigated [17,18]. Zinc-based materials, especially ZnO remains one of the best material for photocatalysis; however, large energy band gap (3.37 eV) and ease of charge carrier recombination limits its application leading to low degradation efficiency of organic compounds [18,19]. As a solution to this limitation, other multifunctional oxide systems have been considered where ZnO has been coupled to additional small band gap semiconductors like Fe_2_O_3_, Cu_2_O, CuO, CdS, and WO_3_ for performance improvement [19]. As other materials continue to be studied as photocatalysts for organic compound degradation, multiple bimetallic tungstate (MWO_4_, M = Mn, Fe, Co, Ni, Cu, Zn) have equally attracted huge interest due to their chemical stability, moderate photocatalytic ability, and post glow photoluminescence properties [20]. Activity efficiency of ZnWO_4_ nanomaterial photocatalysts has been reported. Zhou and et al. reported that applying solvothermal method tuned the morphology of ZnWO_4_ to different sizes and shapes ranging from about ~20 nm nanocrystals to ~35 nm nanorods through the adjustment of ethylene glycol to water solvent ratio with improved resultant effect on the photocatalytic degradation of rhodamine B [21]. Recently, Bi_2_WO_6_/ZnWO_4_ composite fabricated by He et al.; outperformed the unmodified ZnWO_4_ in the photocatalytic degradation of rhodamine dye under UV irradiation [22]. Osotsi et al. reported that 20 mg/L tetracycline was efficiently degraded in the UV and UV-vis-NIR region with over 80% removal employing their fabricated oxygen deficient zinc tungstate nanorods (ZnWO_4−x_) [17]. Bai et al. prepared hybridized graphene modified ZnWO_4_ photocatalyst for the degradation of methylene blue under both UV and visible light irradiation [23]. Ag/AgBr decorated ZnWO4 nanorods improved the degradation of AR18 dye by about 91% in comparison to undecorated forms of ZnWO_4_, TiO_2_, and AgBr as reported by Li et al. [24]. Tian et al. prepared Bi_2_WO_6_ nanosheets with oxygen vacancies using hydrothermal precipitation, demonstrating their high photocatalytic efficiency both in the UV and near infrared (NIR) wavelength for the degradation of methylene orange dye as compared to the pure form [25]. In other study, Sun et al. highlighted by first principle density functional theory (DFT) calculations, upgraded photocatalytic activity of ZnWO_4_ via pairwise co-doping with C, N and F [26]. From studies reported so far, metal/nonmetal doping as a means of improving the photocatalytic activity of ZnWO_4_ has been the focus notwithstanding the possible adoption of photosensitization as modification option as well.

To the best of our knowledge, phthalocyanine based sensitization of ZnWO_4_ as a means of improving photocatalytic ability of the material, has not been documented.

We report for the first-time manganese (III) phthalocyanine (MnPc) sensitized ZnWO_4_ that is (ZnWO_4_MnPc) photocatalyst material for the degradation of bisphenol A (BPA) under 365 nm UV and its extended potential application to the visible light region of 450 nm.

## 2. Materials and Methods

### 2.1. Chemicals

Zn(NO_3_)_2_.6H_2_O, Na_2_WO_4_.2H_2_O were used as precursors of zinc and tungstate oxide, respectively. Manganese (III) phthalocyanine, 1,3-propanesultone (>99%), Dimethylformamide (DMF) (>99%), Dichloro methane (CH_2_Cl_2_) (>99%), bisphenol A (2,2-bis (4-hydroxyphenyl) propane salt (>99%), NaNO_3_, Na_2_CO_3_, NaCl, AlCl_3_, CaCl_2_, KCl, and sodium humic acid, all of high purity (>99%) and p-benzoquinone (BQ) (≥98%), isopropyl alcohol (IPA) (≥99%), Ethylenediamine tetraacetic acid disodium salt dehydrate (EDTA) (≥99%), hydrogen peroxide (H_2_O_2_) (30% *v*/*v*), hydrochloric acid (HCl) (37% *wt.*/*v*), sodium hydroxide (NaOH) (≥99%) were used as purchased from Sigma Aldrich (Darmstadt, Germany) without any further treatment. Mili-Q water has been used for the entire photocatalytic experimental studies.

### 2.2. Instruments

IR spectra were recorded on a Perkin Elmer 1600 FT-IR Spectrophotometer, using KBr pellets. MALDI-MS of the phthalocyanine complexes were obtained in dihydroxybenzoic acid as MALDI matrix using nitrogen laser accumulating 50 laser shots using Bruker Microflex LT MALDI-TOF mass spectrometer (Bremen, Germany). Optical spectra in the UV-vis region were recorded with a Perkin Elmer Lambda 25 spectrophotometer.

X-ray diffraction (XRD) measurement (RIGAKU Corp., Tokyo, Japan) was carried out using a Rigaku D/Max-IIIC diffractometer with CuK_α_ radiation (λ = 0.1541 nm) over the range 2θ = 10°–70° at room temperature, operated at 35 kV and 25 mA at the rate of 3°/min scan speed.

Morphology and microstructure of the synthesized materials was determined with transmission electron microscopy (TEM) employing FEITecnaiG2 Spirit (FEI, Hillsboro, OR, USA), and scanning electron microscopy (SEM) ZEISS EVO LS10 (Carl Zeiss Microscopy, Hamburg, Germany), of 0.2–30 kV acceleration voltage and 0.5 pA–5 µA probe current, coupled to energy dispersive spectroscopy (EDX) detector (Carl Zeiss SmartEDX, Hamburg, Germany), with samples attached to sample holder and coated with gold using sputter-coating (SEM Coating System Machine), while specific surface area was measured with Brunauer–Emmett–Teller (BET) technique recorded on a Micromeritics ASAP 2010 System (Micromeritics, Norcross, GA, USA), with all samples outgassed under vacuum (residual pressure 10^−2^ mbar) in order to eliminate gaseous contaminants. Pore size analysis was obtained by applying the Barrett, Joyner, and Halenda (BJH) method on the adsorption branch of the isotherms to ascertain the type IV mesoporousity of the materials.

X-ray photoelectron spectroscopy (XPS) data for the chemical environment revelation of the materials was obtained using a PHI 5000 VersaProbe Spectrometer (ULVAC-PHI, Inc.; Kanagawa, Japan), with an AlK_α_ radiation source.

### 2.3. Preparation and Characterization of Photocatalyst Materials

#### 2.3.1. ZnWO_4_

ZnWO_4_ was prepared by hydrothermal method according to literature [27].

Namely, equimolar (0.1 M) amounts of Zn (NO_3_)_2_. 6H_2_O and Na_2_WO_4_. 2H_2_O were dissolved in 25 mL of water. Mixture solution was magnetically agitated and vigorously for 15 min during which time obtained white solution mixture was subsequently transferred to an autoclave heated at a temperature of 150 °C for 5 h and cooled to room temperature, settled for 2 h; after which the supernatant liquid phase was decanted. The precipitate dried off on hot plate at 85 °C for a day and grounded into powder as ZnWO_4_ and used throughout the experiment.

#### 2.3.2. ZnWO_4_MnPc

- Synthesis and Characterization of Zwitter-Ionic Water-Soluble Manganese (III) Phthalocyanine (BZ-Mn-PS)

**Scheme 1:**
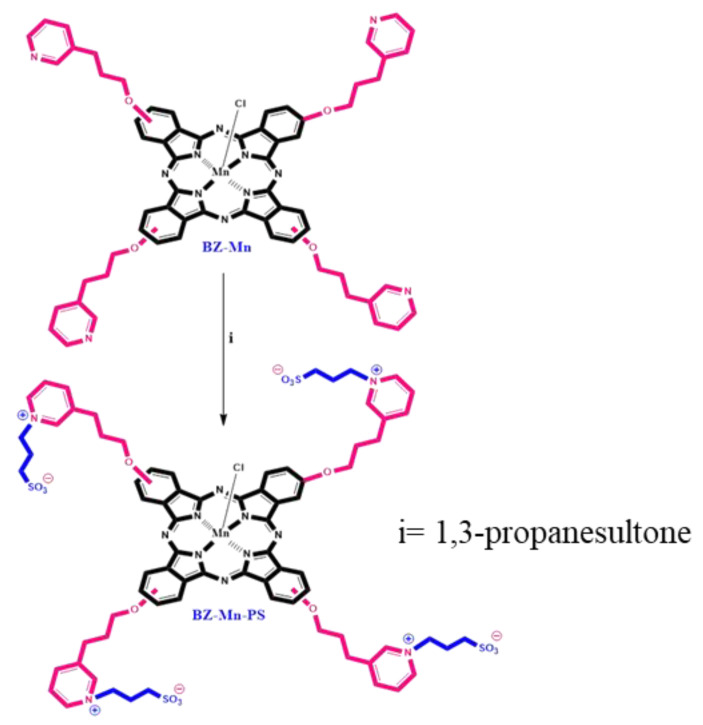
Synthesis route of BZ-Mn-PS.

The benzene ring zwitterionic water-soluble manganese (III) phthalocyanine molecule complex herein referred as (BZ-Mn-PS) was synthesized according to literature [28] as shown in Scheme 1.

Manganese (III) phthalocyanine (60 mg, 0.052 mmol) and excess 1,3-propanesultone were dissolved in DMF (5 mL) and the reaction mixture was stirred at 70 °C for 24 h under nitrogen atmosphere. Then, the reaction mixture was cooled to room temperature and the product was precipitated by the addition of CH_2_Cl_2_. The precipitate was filtered off and washed with acetone to remove unreacted 1,3-propanesultone. Yield: 75 mg (91%) with m.p. >300 °C. Elemental Analysis: IR (ATR), ν/cm^−1^ found: 3058 (Aromatic-CH), 2940–2860 (Aliphatic C-H), 1603, 1503, 1487, 1461, 1395, 1340, 1288, 1177, 1133, 1076, 1054, 1030, 963, 877, 821, 790, 747, 687. MALDI-TOF-MS m/z calc. 1632.17; found: 408.05 [M]^+4^. UV-Vis (DMSO) λ_max_ nm (log *ε*): 732 (5.02), 658 (4.39), 501 (4.37), 369 (4.73).

1 wt.% of BZ-Mn-PS was loaded in ZnWO_4_ powder material in situ via hydrothermal autoclaving to obtain what is from hence forward referred to as manganese phthalocyanine sensitized ZnWO_4_ material (ZnWO_4_MnPc) and employed throughout the experimental studies with ZnWO_4_ material designated as control.

### 2.4. Determination of pH_pzc_ of ZnWO_4_MnPc Catalyst Material

To determine pH point of zero charge (pH_pzc_), drift method was employed [29]. Namely, 50 mL of 0.01 M NaCl solution was measured out in conical flasks. The initial pH values of these solutions were adjusted and stabilized at room temperature to values between 2 and 12 using either HCl or NaOH of 1M concentration each. Upon stability of the initial pH values, 0.05 g powder of the synthesized catalyst (ZnWO_4_MnPc) was added to each of the measured flask solutions, stirred for 48 h and final pH value of each flask solution measured using HANNA edge digital pH meter (model HI2020).

### 2.5. Photocatalytic Experiments

The photocatalytic performance of the synthesized materials was evaluated towards the degradation of BPA under 365 nm irradiation and also tested in the perspective of future work under visible light source of 450 nm wavelength. An internal 200 degree reflector for optimal efficiency (99.9%), UV-A and visible blue wavelength in the range of 300 nm to 475 nm spectral power distribution was fabricated inhouse with 5 Philips Mecury (Hg) lamps–TL-K 40W/10R ACTINIC BL REFLECTOR, (Germany) and employed for the photocatalytic tests of the synthesized materials. The intensity of the light source measured with solar power meter Lafayette SPM-7, (Italy) was 1.2 mW/cm^2^ and 2.3 mW/cm^2^ for the UV 365 nm and extended visible 450 nm cut off filter light source wavelengths respectively

Forty milliliters of working suspension containing 1g/L catalyst and 10 mg/L of BPA was introduced into 50 mL cylindrically shaped quartz glass sleeve reactor cells and kept under mechanical agitation during the irradiation. Prior to irradiation, suspension was stirred in the dark for 30 min to establish adsorption–desorption equilibrium while irradiation stability was achieved. Outside the natural pH values of the catalysts working suspensions, pH values of 3, 5, 9, and 11 were tested and finally all other experimental tests carried out at optimum pH 11. A distance of 30 cm between the sample and the light source was set. At chosen time intervals, about 1 mL sample aliquots were drawn from reactor cells, cooled and filtered through 0.45 µm CA filter for HPLC analysis for monitoring of BPA degradation on Kinetex 5 µm EVO C18, column 150 mm × 4.6 mm Phenomenex (USA). Runtime was 15 min and eluents were acidic 40% MilliQ water (H_3_PO_4_, pH ~3) and 60% acetonitrile with a flow rate of 0.2 mL.min^−1^. BPA quantification was performed at 230 nm wavelength with a retention time of ~10 min.

The photocatalytic degradation process of BPA follows a pseudo first order reaction kinetic, reaction rate of BPA was estimated as a linear regression slope according to expressed Equation (1):

−ln C/C_0_ = k_app_t (1)

where, k_app_, C_0_, and C are apparent degradation rate constant, initial concentration, and concentration after time t, respectively. All photocatalytic degradation experimental data were average value of triplicate measurements.

## 3. Results and Discussion

### 3.1. Material Characterizations

#### 3.1.1. Synthesis and Characterization of BZ-Mn-PS

**Figure 1:**
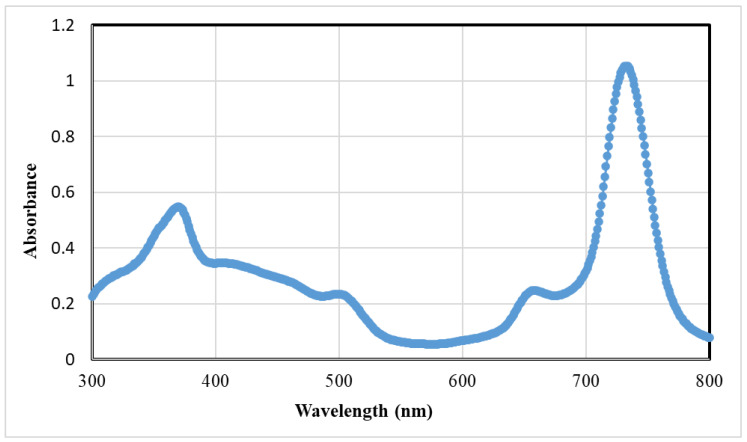
UV-Vis spectrum in DMSO of BZ-Mn-PS.

The synthetic route of the zwitterionic water-soluble manganese (III) phthalocyanine BZ-Mn-PS was given in Scheme 1. Zwitterionic water-soluble manganese (III) phthalocyanine BZ-Mn-PS was synthesized from BZ-Mn phthalocyanine by the ring opening reaction of 1,3-propanesultone (i) in DMF at 70 °C. The FT-IR spectrum of BZ-Mn-PS, Figure S1 of supp. Section, indicated stretching of aromatic C-H at 3058 cm^−1^, aliphatic C-H at 2940–2860 cm^−1^, respectively. The vibration peaks observed at 1340 cm^−1^ (asymmetric S=O vibration for sulfobetaine) and 1160 cm^−1^ (symmetric S=O vibration for sulfobetaine) were indicative for the conversion of the starting BZ-Mn phthalocyanine to sulfobetaine derivative BZ-Mn-PS. In the MALDI-TOF mass spectra, supp. info of Figure S2, BZ-Mn-PS molecular ion peak was observed at m/z: 408.05 as (M + 4)/4 because of multiple charged positive ion for [M]^+4^ [30]. The UV-Vis absorption spectrum of BZ-Mn-PS in DMSO was shown in Figure 1. UV-Vis spectra of BZ-Mn-PS exhibited intense single Q band absorption of π/π* transition at 732 nm with the B band observed in the UV region at 369 nm. These two peaks of B and Q bands are in general agreement with reported literature values of phthalocyanine derivative absorption at 350 nm and 670 nm respectively. The Q band represents π→π* transition between highest occupied molecular orbital (HOMO) and lowest unoccupied molecular orbital (LUMO) as well as the transition from deeper level π orbital to LUMO by the B band [31]. Near the Q band, similar vibrational band peaks to the peaks observed at 658 nm and at 501 nm (Figure 1), have been reported and attributed to metal to ligand charge transfers in manganese phthalocyanines [32].

#### 3.1.2. Characterization of ZnWO_4_ and ZnWO_4_MnPc

**Figure 2:**
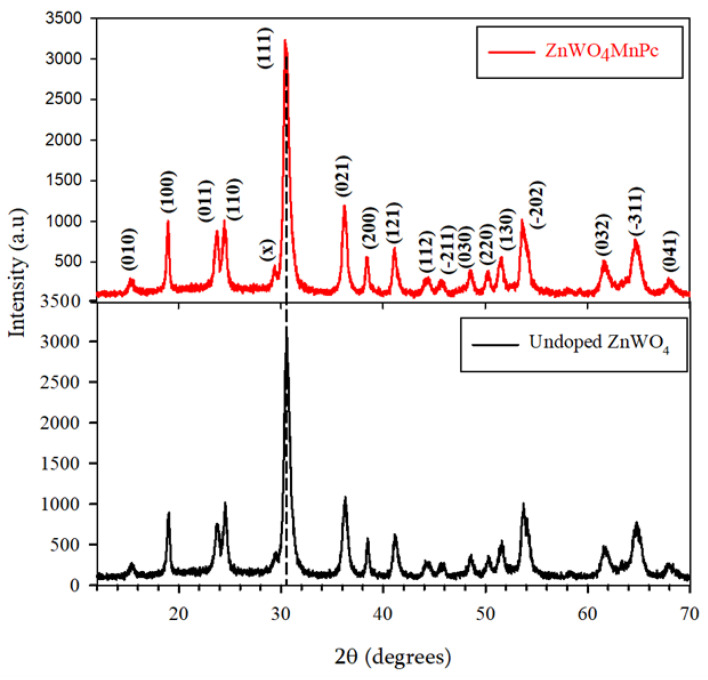
XRD patterns of ZnWO_4_ and ZnWO_4_MnPc sample materials.

Crystalline phase structure of the materials was determined by XRD and as can be observed from Figure 2, the loading of the ZnWO_4_ with MnPc did not make any significant change in the crystallinity of the material. The pure phase monoclinic sanmartinite ZnWO_4_ structure with a P_2/_c space group according to the standard reference data (JCPDS # 00-015-0774) is identified in both synthesized materials. As can be seen from Figure 2, the diffraction pattern of ZnWO_4_MnPc material obtained after Pc addition in ZnWO_4_ showed a slightly increased FWHM, i.e., improved crystallinity of the (111) plane of ZnWO_4_MnPc at 2θ of 30.45° over that of ZnWO_4_ [20,33,34]. In addition, an unidentified small peak (**X**) is found at the base of the (111) plane peak of ZnWO_4_MnPc. This peak is less intense in undoped ZnWO_4_. The calculated crystallite sizes of synthesized materials are reported in Table 1. Values were obtained using the Debye Scherrer relationship as expressed in Equation (2).

D_hkl_ = Kλ/β*_hkl_*cosθ (2)

where, λ = 1.54059 Å is the wavelength of the CuK_α_ source, β is the integral breadth of the XRD peaks depending on the width of the particular *hkl* plane and taken at full width at half height maximum (FWHM) in degrees, θ is the diffraction angle obtained from the XRD data of the strongest reflection and K is the shape factor kept at constant value of 0.9.

The crystallite size of Pc doped ZnWO_4_ material was a bit larger at 15.1 nm as compared to the undoped ZnWO_4_ sample material of crystallite size 13.1 nm and very much in agreement with reported literature data of 13 nm [34]. The slight increase in crystallite size in ZnWO_4_ phase of ZnWO_4_MnPc at 15.1 nm (Table 1) over ZnWO_4_ material was not only due to the additional hydrothermal treatment for the incorporation of Pc, but also the large nature of the Pc molecules.

**Figure 3:**
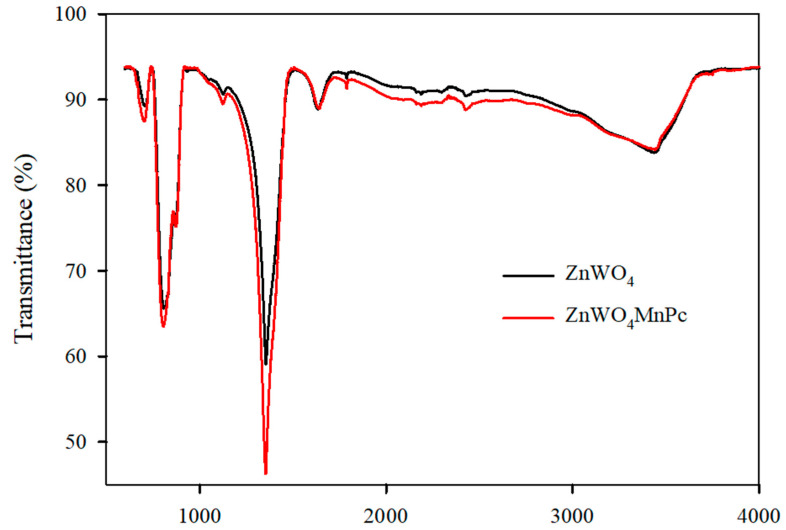
FTIR patterns of ZnWO_4_ and ZnWO_4_MnPc sample materials.

In the FT-IR spectroscopy results, the recorded peaks in the range 750 to 3450 cm^−1^ are in agreement with literature data for ZnWO_4_ [34] (Figure 3). FTIR spectra of Figure 3 display associated absorption peaks of ZnWO_4_ and ZnWO_4_MnPc materials around 706 cm^−1^, which have been reported and attributed to the bending and stretching absorption bands of Zn–O and W–O bonds. Peaks at around 806 cm^−1^ and 872 cm^−1^ have been due to bending and stretching of vibrational bonds of Zn-W–O bonds present in the synthesized materials [20,23,31,33,34,35,36]. The intense peak observed at around 1350 cm^−1^ is due to typical surface hydroxyl groups [35,37,38,39]. Peak at approximately 2200 cm^−1^ is attributed to –CH_2_ functional group vibrations, the ones at around 1600 cm^−1^ and 3400 cm^−1^ are characteristic of bending and stretching of the hydrogen bond of surface absorbed physical water (H–O–H ) and its corresponding O–H bond stretching, respectively [40].

**Figure 4:**
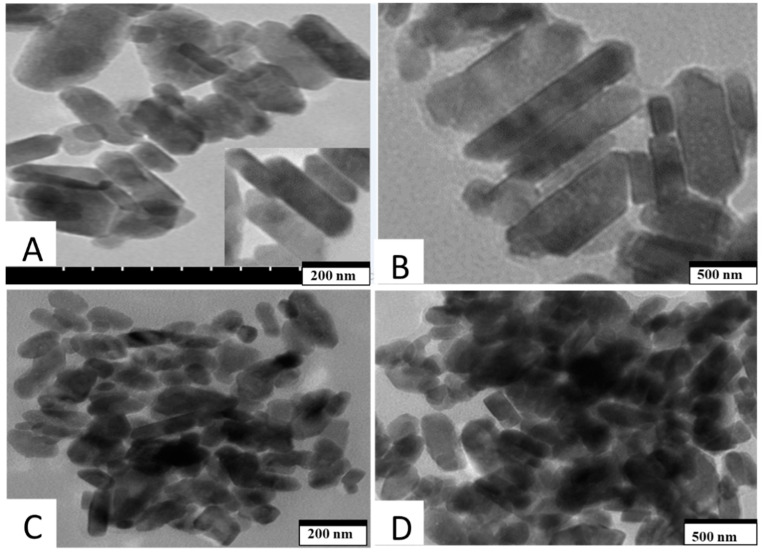
TEM images of as synthesized photocatalyst materials: ZnWO_4_ (**A**,**B**), and ZnWO_4_MnPc (**C**,**D**).

TEM images of the synthesized samples (Figure 4), show the interesting nano particle morphology of both ZnWO_4_MnPc and ZnWO_4_ materials. The TEM image morphology and microstructures of the prepared photocatalysts materials show, that ZnWO_4_ and ZnWO_4_MnPc nanoparticles were similar in shape and approximately hexagonal shaped rods or elongated polyhedral (Figure 4A–D) A rather uniform size distribution is observed in the case of ZnWO_4_ (Figure 4A,B). Similar shape but slightly larger and rather aggregated nanoparticles are found for ZnWO_4_MnPc most probably due to the surface functionalization of ZnWO_4_ by the MnPc molecules (Figure 4C,D). The obtained average particle size values from TEM measurements are displayed in Table 1, as 13.4 nm for ZnWO_4_ and 20.3 nm for ZnWO_4_MnPc material. These results are very close with the primary crystallite size calculated by the XRD data with a value of 13.1 nm for ZnWO_4_ (Table 2) and 15.1 nm for ZnWO_4_MnPc. Considering the polycrystalline nature of the prepared materials, there is a good agreement among the XRD and TEM derived data (Table 1, Figure 2, and Figure 4A–D) [18].

**Figure 5:**
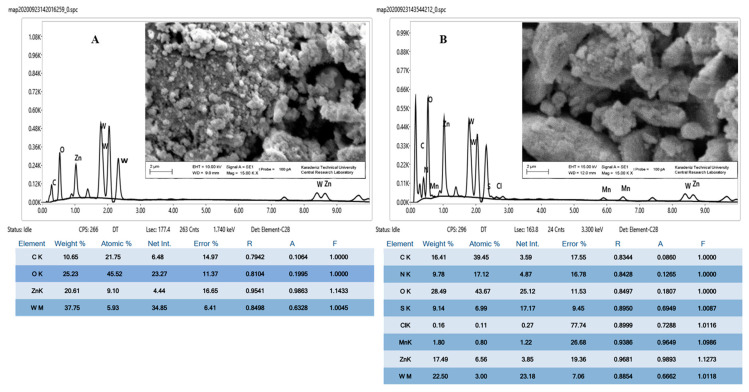
SEM images and associated EDX maps of as synthesized photocatalyst materials: (**A**) ZnWO_4_, and (**B**) ZnWO_4_MnPc.

In order to visualize the surface structural morphology and identify associated material elemental compositions of the synthesized materials, scanning electron microscopy (SEM) in connection with energy dispersive x-ray spectroscopy measurement (EDX), was carried out. As can be seen from Figure 5, ZnWO_4_ and ZnWO_4_MnPc consist of irregularly shaped particulates. However, a more agglomerated nature is revealed for ZnWO_4_MnPc material. Again, this can be attributed to the added phthalocyanine molecule (MnPc), (Figure 5B). On the other hand, EDX analysis confirmed the elemental composition and indicated the existence of MnPc doping (Figure 5). Lack of N, S, Cl, and Mn is recorded for ZnWO_4_. In contrary such elements exist in low concentration in ZnWO_4_MnPc confirming the successful MnPc doping on ZnWO_4_.

**Figure 6:**
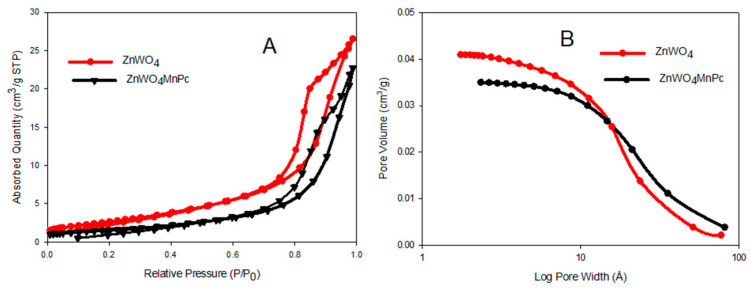
N_2_ adsorption–desorption isotherms (**A**), and adsorption cumulative distribution of synthesized materials (**B**).

N_2_ adsorption–desorption isotherms of the materials are shown in Figure 6A. Isotherms are type IV with H3 hysteresis loop indicating the mesoporous nature of the materials [41,42]. BET surface area as measured (Table 1) for ZnWO_4_MnPc in comparison to ZnWO_4_ material at a value of 11.4 m^2^/g was lower than that of the undoped ZnWO_4_ material with 15.1 m^2^/g. This small reduction in BET surface area after the Pc sensitization of ZnWO_4_ is due to surface coverage of the latter by Pc molecules towards improved interaction for the desired improved photoactivity [35]. The values of total pore volume (V_p_) of ZnWO_4_ and ZnWO_4_MnPc materials follow the same trend with respective values of 0.04 cm^3^/g and 0.035 cm^3^/g, while average pore size have respective values of 14 nm and 19 nm.

**Figure 7:**
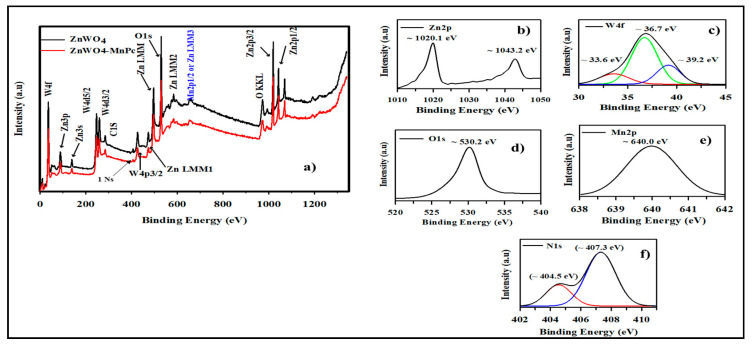
(**a**) XPS survey spectrum of ZnWO_4_ and ZnWO_4_MnPc synthesized materials, and (**b**) Zn2p, (**c**) W4f, (**d**) O1s, (**e**) Mn2p, and (**f**) N 1s deconvoluted peaks of synthesized materials.

XPS measurement depicts the chemical environments of synthesized materials as be seen from Figure 7. The XPS survey and elemental spectra of ZnWO_4_ sample and its MnPc sensitized analogue is given in Figure 7a. Surface elemental chemical components of both sample materials were identified with Zn, W, O, and Mn being predominant. Nitrogen of the phthalocyanine component of ZnWO_4_MnPc material was detected though a small peak. XPS results (Figure 7b) showed Zn 2p_3/2_ and Zn 2p_1/2_ peaks resolved at high resolution scan of binding energy ~1020.1 eV and ~1043.2 eV, respectively and were in agreement with literature [43,44,45,46,47]. Spectrum in Figure 7c revealed high resolution peak of W 4f with 4f_7/2_ and 4f_5/2_ peaks appearing at 33.6 eV and 36.7 eV respectively; and attributed to W–O bond. The small shift of a peak to the higher binding energy of 39.2 eV, is attributed also to part of the W^5+^/W^+6^ valence states of W 4f_5/2_ and W 4f_7/2_ spin–orbit doublets. This value is approximately the same with previously reported (Figure 7c) [48]. As can be seen in Figure 7d, XPS spectrum indicated peak of O 1s at a typical binding energy of 530.2 eV [43,44,45,46,47]. In Figure 7e, f XPS spectra, the binding energy of Mn2p_3/2_ was found at 640 eV and matches the binding energy of Mn^2+^ in MnO. This is a further indication of the divalent state of manganese in the Pc, prone for redox reactions and successful substitution confirmation of same at the tetrahedral sites of the ZnWO_4_ material surrounded by the O^2-^ ions [49]. N 1s was recorded at 404. 5 eV accompanied by a sharper higher BE peak at 407. 3 eV further confirming Pc presence. Though, the resolved peaks of Zn 2p_3/2_, Zn 2p_1/2_, W 4f_7/2_, and W 4f_5/2_ were all registered at binding energy values in good agreement with values reported in literature. They differ with respect to host ZnWO_4_ material due to their shift to lower binding energy. Observed peak show a shift of Zn 2p_3/2_, Zn 2p_1/2_, W 4f_7/2_, and W 4f_5/2_ that can be attributed to a distortion or variation in their physical or chemical environment. Zn, O and W element of the host ZnWO_4_ material are found of modified chemical state and/or local bonding after Pc modification [43,44,45,46,47].

**Figure 8:**
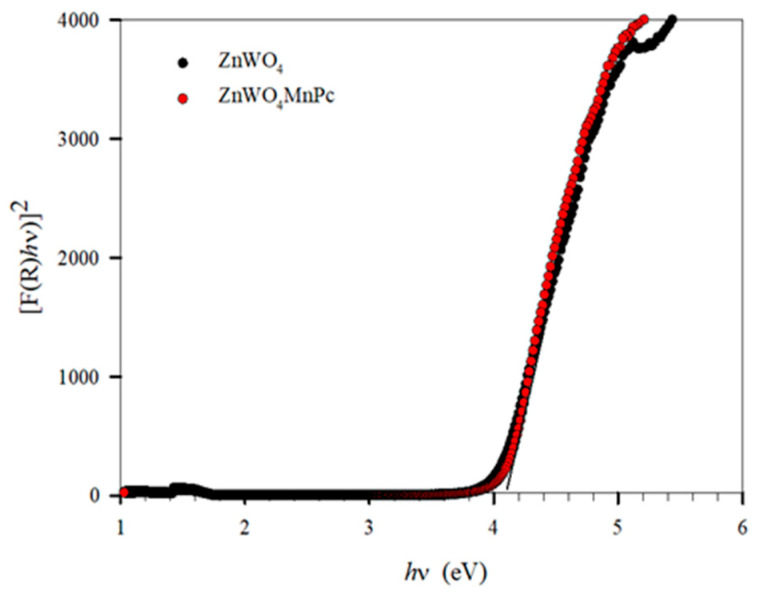
Energy band gap (E_g_) of synthesized materials.

Optical energy band gaps of the photocatalyst materials were estimated by UV-vis diffuse reflectance spectroscopy (DRS). Figure 8 shows the Tauc plot from Kubelka–Munk function (Equation (3)) used to transform the reflectance spectra into absorption spectra [50]. Band gap energies of the materials as estimated from constructed Tauc plots of hν vs. [F(R)hν)]^2^ and extrapolating the linear part to [F(R)hν)]^2^ equal to zero as can be seen from Figure 8 was 4 eV for ZnWO_4_ and 4.1 eV for ZnWO_4_MnPc (see Table 1). The related UV-vis/DRS measurement for the two materials, also showed similar reflectance as depicted in Figure S3 of the supp. section, hence their approximate energy band gap (E_g_) values.

F(R) = S(1 − R)^2^/2R (3)

where F(R), R and S are absorbance, reflectivity, and scattering factor, respectively. In our case, we have assumed our S factor to be a unit since our materials are in powder form rather than film. Although a variation within the range 3.9–4.4 eV is observed in literature results regarding the energy band gaps of bivalent metal tungstates, our values are well within this range [51]. For the band gap energy (4.1 eV) of ZnWO_4_MnPc, there is a slight red shift of 0.1 eV to higher energy level. A slight shift to higher band gap energies in the case of semiconductor oxide type materials, i.e., ZnO, CeO_2_, etc. is related to particle size features and has been attributed to quantum size effects [52]. Energy band shifts in various oxide nanocrystals, is also related to factors like bulk or surface doping effects and lattice structural modifications [44,52,53]. However, no such an effect is expected by the loading of Pc to ZnWO_4_. Connecting all these information, with respect to the energy band gaps of the synthesized materials; their insignificant red shift in binding energy points to the fact that the surface Pc sensitization of ZnWO_4_ did not to a noticeable extent interfere with sample in the introduction of defects and subsequent modification of sample local lattice symmetry which could have pulled off changes in band structural properties. Hence, a red shift in energy band gap of our samples compared to band gap energy already investigated for bulk ZnWO_4_ in the range 3.9–4.4 eV [51] was not possible.

### 3.2. Photocatalytic Activity

#### 3.2.1. Effect of pH

- pHpzc Value

**Figure 9:**
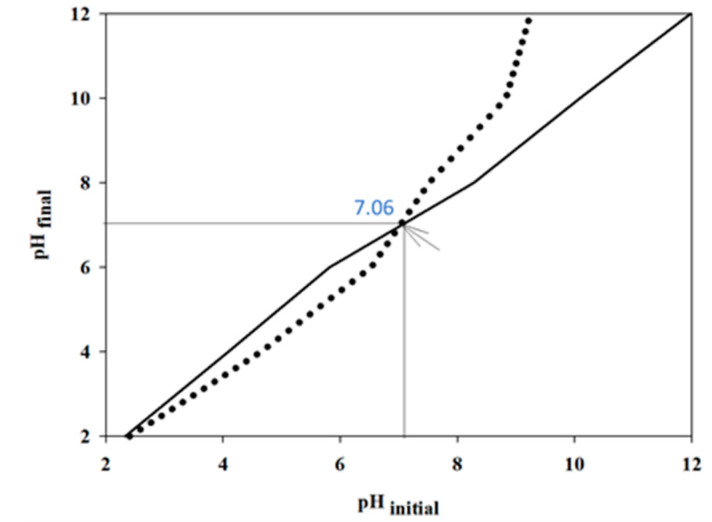
Plot for the determination of pH point of zero charge (pH_pzc_) of ZnWO_4_MnPc photocatalyst.

**Figure 10:**
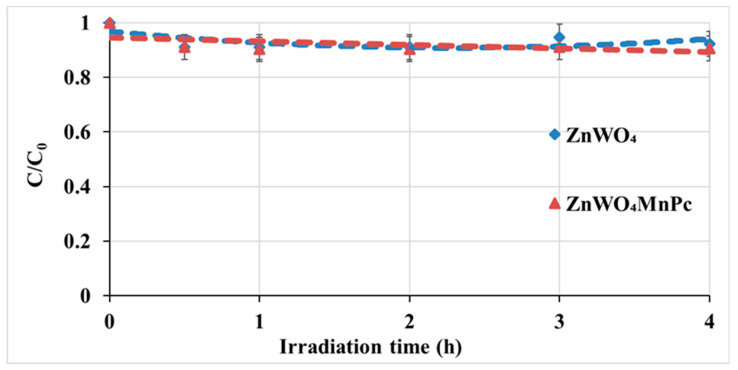
ZnWO_4_ (blue line), and ZnWO_4_MnPc (red line) photocatalyst materials degradation towards BPA. ([BPA]_0_ = 10 mg/L, Catalyst dosage = 1 g, ZnWO_4_ pH_natural_ = 6.58, and ZnWO_4_MnPc pH_natural_ = 6.68).

**Figure 11:**
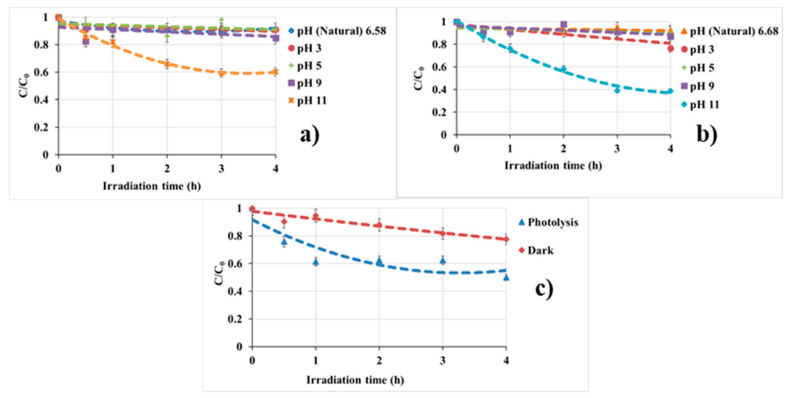
Initial pH effects on (**a**) ZnWO_4_, and (**b**) ZnWO_4_MnPc photocatalyst materials towards BPA degradation, and (**c**) photolysis and dark tests ([BPA]_0_ = 10 mg/L, Catalyst dosage = 1 g).

pH is an important parameter in the degradation process of organic chemical compounds. Thus, in order to investigate pH effect on the photodegradation efficiency of ZnWO_4_MnPc, its pH_pzc_ was determined and found 7.06 (Figure 9). This shift to high pH_pzc_ of ZnWO_4_MnPc might be attributed to the presence of the electron rich Pc molecule in the composite material. Initially, at a system, non-modified pH values of 6.58 and 6.68 were measured for ZnWO_4_ and ZnWO_4_MnPc aqueous suspensions, respectively. A 1 g/L catalyst was used for each experiment against 10 mg/L of initial BPA concentration. These conditions showed no significant BPA degradation after 4 h of 365 nm UV irradiation as displayed in Figure 10. Thereafter, pH values of 3, 5, 9, and 11 were also tested (Figure 11) At a highly alkaline pH 11 and under same conditions of catalyst and initial BPA concentration, ZnWO_4_MnPc achieved about 60% of BPA degradation after 4 h irradiation while ZnWO_4_ degraded only 40% in the same time. It has been reported that enhanced BPA degradation is observed in high pH values [54]. The observed trend in the initial pH effect on the photocatalytic efficiency of ZnWO_4_MnPc towards BPA removal can be correlated with the ionization of BPA molecule which within its pK_a1_ and pK_a2_ reported respective range values of about 9.59 and 11.30 dissociate and exist in aqueous systems as mono BPA^−^ or divalent BPA^2**−**^ anions [53,55,56]. At tested pH 9 and pH 11, BPA molecule will be deprotonated based on reported pK_a_ value [53,57]. Therefore, at high alkaline pH 11, with respect to the pK_a_ value and in connection with pH_pzc_ of ZnWO_4_MnPc, there will be no predominance of electrostatic force of attraction to attract BPA deprotonated anions onto the active sites of the negatively charged catalyst surface; however, reported surface adsorption mechanism of BPA molecule at increased pH above 7 as well as the dominance of BPA^2**−**^ dianion reported highest at pH 11within the pH range of 8–12, will be primarily responsible for the enhanced adsorption of BPA onto the catalyst surface for subsequent degradation [53,57]. On the contrary, at pH 9, the catalyst surface will remain negatively charged based also on the pH_pzc_. However, at pH 9, within the range of reported BPA pKa value, BPA**^−^** exits but, majority of the molecule remain undissociated and its neutral form still dominant [53,57]. Hence, the reason why at pH 9, there was no significant BPA removal as basically there was little or lack of electrostatic attraction and surface adsorption mechanism made little impact [53,57]. Similarly, at initial pH 3, (the unmodified natural pH 6.68 for ZnWO_4_MnPc catalyst suspension), and pH 5, the surface of the catalyst based on the 7.06 pH_pzc_ will be positively charged as well as protonated BPA molecules based on the pKa value 9.59–11.30 [53,57]. Hence, the observed negligible or even non-BPA removal at these pH values due to like charge force repulsions (Figure 11). Also, the reported low octanol-water partition coefficient (log Kow) range of BPA (2.20–3.82) predicted its undissociation at acidic pH and low solubility especially at typical ambient pH values of 6–8 [56]. All these information, highlight further why in our system, there was little or no BPA removal under the investigated initial pH values of 3–9. Outside attributing the possible BPA disappearance at pH 11 for reason of change of surface properties of the investigated photocatalyst material, BPA removal from heavy BPA laden water has been reported achieved with TAML/peroxide catalyst which showed outstanding efficacy with greater than 99% BPA reduction in 15 min [57]. Going further, all other experiments were carried out at pH 11 with results for tested parameters.

For control experiments, Photolysis tests without any catalyst were performed as control experiments to check the degradation of BPA. BPA degradation was also followed in the presence of catalyst under dark conditions. No BPA degradation was achieved during 4 h of the dark test (Figure 11c). Photolysis experiment showed almost 40% of BPA removed after 4 h of irradiation under 365 nm. It has been reported that BPA exhibits strong absorption of UV light wavelengths of more than 290 nm of sunlight in basic solution conditions [56]. However, extent of removal depends on pH, turbidity, turbulence as well as light intensity [56].

#### 3.2.2. Effect of Catalyst Dosage and Initial BPA Concentration

**Figure 12:**
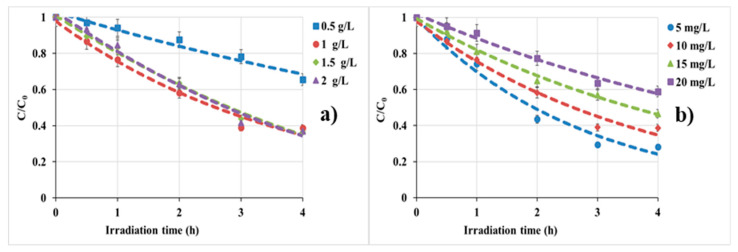
Effects of (**a**) ZnWO_4_MnPc catalyst dose against 10 mg/L BPA concentration, and (**b**) BPA concentrations against 1 g catalyst dose on photocatalytic system at pH 11.

To investigate the effect of catalyst dose, experiments were performed with different amounts of the ZnWO_4_MnPc photocatalyst. In addition to 1 g/L catalyst dose, 0.5 g/L, 1.5 g/L, and 2 g/L doses of ZnWO_4_MnPc were investigated of their photoactivity at pH 11 towards 10 mg/L BPA degradation. As observed from Figure 12, at 0.5 g catalyst dose, only about 34% was removed. This was about half of what removed at the same time (4 h) of irradiation at 365 nm with 1 g/L, 1.5 g/L, and 2 g/L. However, among larger catalyst doses the optimum was 1 g/L as any further increase i.e.,1.5 g/L and 2 g/L did not reflect any additional significance in BPA removal (Figure 12a).

The effect of initial BPA concentration on the catalyst performance was also investigated. As can be seen from Figure 12b, in addition to 10 mg/L BPA, other concentrations of 5 mg/L, 15 mg/L, and 20 mg/L were tested. After 4 h irradiation the removal rate diminishes at higher BPA concentration. BPA or its degradation products are expected to saturate the catalyst surface in a non-reversible way reducing the overall activity of the catalyst [58]. The best response was for 1 g/L ZnWO_4_MnPc exposed to 5 mg/L BPA (Figure 12b). However, to ensure that minimum amount of catalyst was used for a considerate BPA concentration, 10 mg/L BPA was deployed for an amount of 1 g/L catalyst dose in this study.

#### 3.2.3. Reactive Oxygen Species (ROS) Probe

**Figure 13:**
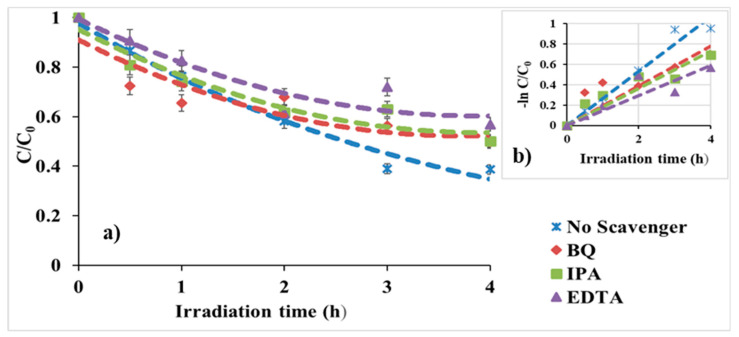
(**a**) Effects of scavengers on BPA removal (1 g ZnWO_4_MnPc, [BPA]_0_ = 10 mg/L, pH 11), and (**b**) corresponding degradation rate constant (inset).

Photocatalytic process has been reported to depend on various reactive species like super oxide radicals (O_2_^−^), hydroxyl radicals (HO**·**) and hydrogen peroxide (H_2_O_2_) which drive the reaction system [59]. Investigation of these reactive oxygen species (ROS) responsible for the photoxidation of BPA was carried out. In this test, benzoquinone (BQ), isopropyl alcohol (IPA), and ethylenediamine acetic acid (EDTA) each of 1 mM concentration were introduced into the photocatalytic reaction system as quenchers to scavenge super oxide radical (O_2_^−^), hydroxyl radical (HO**·**) and holes (h^+^), respectively. As can be seen in Figure 13, all three quenchers exerted influence on the photodegradation rate of the catalyst during the 4 h irradiation time. Upon the addition of BQ, over 60% of BPA removed after 4 h irradiation time with a photodegradation rate of 0.2661 h^−1^, (Table 2) in the absence of scavenger dropped to 0.1942 h^−1^ for just 40% of BPA removed. In the case of IPA, a similar effect to that of BQ was recorded. Photodegradation rate of the catalyst reduced to 0.1820 h^−1^. On the other hand, a more enhanced influence was obtained by EDTA. In the latter case photodegradation rate reduced to 0.1474 h^−1^. This is about half the rate measured without any scavenger (Table 2), to just 30% of BPA removed. Thus, superoxide anion (O_2_^−^), hydroxyl radical (HO**·**), and holes (h^+^) reactive species were all involved in the degradation of BPA, and with significant contribution coming from generated hole carriers (h^+^). Therefore, based on these findings a reaction mechanism for the degradation of BPA in this study may follow such a pathway where the conventional (O_2_^−^), (HO**·**), and holes (h^+^) reactive oxygen species (ROS) are expected to participate.

#### 3.2.4. Effect of Hydrogen Peroxide (H_2_O_2_)

**Figure 14:**
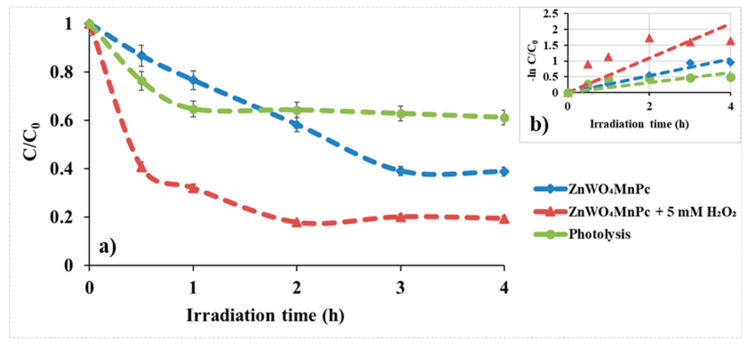
(**a**) Effect of H_2_O_2_, and photolysis on BPA removal (1 g ZnWO_4_MnPc, [BPA]_0_ = 10 mg/L, pH 11), and (**b**) corresponding degradation rate constant (inset).

The influence of H_2_O_2_ on the degradation of BPA during the UV/ZnWO_4_MnPc process was investigated. A quantity of 5 mM H_2_O_2_ was selected to be added to the photocatalytic reaction system as a suitable dose in order to avoid excessive scavenge of electron (e^−^) and hydroxyl (HO**·**) radical and consequently decrease in the degradation rate [54,60]. The addition of 5 mM H_2_O_2_ resulted in about 80% of BPA removed after 4 h irradiation. This is higher compared to 60% of BPA removal in the absence of H_2_O_2_ (Figure 14). The respective degradation rate constant of the catalyst in the presence of H_2_O_2_ (0.5442 h^−1^) is more than double of that without H_2_O_2_ at (0.2661 h^−1^) (Figure 14b). The improvement of the photocatalytic degradation performance can be attributed to the formation of additional reactive species with subsequent suppression of the recombination of electron–hole charge carriers [54]. On the other hand, influence of photolysis on the degradation rate of BPA where at 4 h irradiation about 40% was removed can be seen from (Figure 14a) displaying a predominant driving force for about 1 h irradiation. At this time, photolysis was on par performance with catalyst, stabilizing thereafter to achieve a degradation rate of 0.1595 h^−1^ in comparison to that of 0.2661 h^−1^ for the catalyst (Table 2) at 4 h irradiation. This scenario, points to the active role of photolysis in driving the photocatalytic process degradation of BPA over the 4 h irradiation [56].

#### 3.2.5. Water Matrix Effects

**Figure 15:**
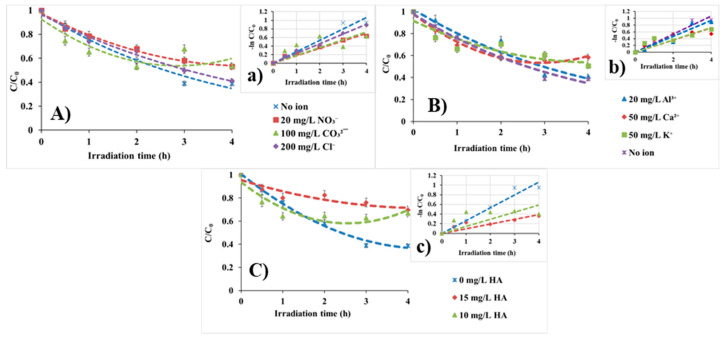
Inorganic ion species effect on BPA removal (1 g ZnWO_4_MnPc, [BPA]_0_ = 10 mg/L, pH 11): (**a**) Anions and rate of degradation (inset), (**b**) cations and rate of degradation (inset), and (**c**) humic acid (HA) and rate of degradation (inset).

Inorganic species are known to influence photocatalytic degradation of organic contaminants [61]. Therefore, to simulate the effect of water matrix on the degradation efficiency of the catalyst, the presence of ions and humic acid effect were investigated. As can be seen from Figure 15a, the addition of 20 mg/L NO_3_^−^, 100 mg/L CO_3_^2−^, and 200 mg/L CI^−^, had significant effect on the degradation process of BPA. 20 mg/L NO_3_^−^, suppressed most BPA removal with a degradation rate constant of 0.1735 h^−1^ and around 40% of BPA removed in comparison to degradation rate constant of 0.2661 h^−1^ with slightly over 60% of BPA removed in same irradiation time when no anion was added. With 100 mg/L CO_3_^2−^ ion added to the reaction system, there was initially a slight improvement in the amount of BPA removed in comparison to no added anion up to 2 h of irradiation. However, no significant difference was observed at these irradiation conditions when compared to no anion addition. However, soon after the first hour a decline in BPA degradation is observed. This behavior is attributed to a mixed role of carbonates under certain conditions of concentrations, pH, pK_a_, etc. that may act in favor of a pollutant degradation or even suppress it while acting as a scavenger for hydroxyl (HO**·**) reactive species [62].

In the case of 200 mg/L Cl^−^, into the reaction system, chloride ions interfered with a minimal effect compared to the nitrates or carbonates. Degradation rate constant was found 0.227 h^−1^ with 200 mg/L Cl¯ in comparison to 0.2661 h^−1^ without any added anion (Figure 15a). Overall, the anions suppression effect in the photocatalytic degradation follows the order: 20 mg/L NO_3_^−^ > 100 mg/L CO_3_^2−^ >200 mg/L Cl^−^. Such a suppression effects is possibly a result of a competitive absorbance of the inorganic ions on the surface of the catalyst whereby rate of photodegradation process is reduced [63].

On the other hand, upon the addition of cations (Figure 15b) both of Ca^2+^ and K^+^, ions at 50 mg/L each caused no significant difference. The same degradation rate of 0.1795 h^−1^ was measured while for no cation addition the degradation rate constant was 0.2661 h^−1^ (Table 1). In contrast, 20 mg/L Al^3+^ ion made the least impact in response to the degradation process in comparison to Ca^2+^ and K^+^. A 0.2336 h^−1^ degradation rate constant was obtained on the addition of 20 mg/L Al^3+^ ion after 4 h irradiation (inset Figure 15b). The photocatalytic process degradation reduction effects upon the addition of the cations was as follows: 50 mg/L Ca^2+^ = 50 mg/L K^+^ >20 mg/L Al^3+^. Generally, all added ions have caused photocatalytic process decline. This finding can be attributed to reported phenomenon of hydroxyl radical scavenging, pH change of reaction solution and blockage of catalyst active sites by adsorption of cationic species [61] (Figure 15a,b, Table 2).

The role of humic acid (HA) on the degradation process, was investigated under 10 mg/L and 15 mg/L HA, respectively (Figure 15c). Both concentrations of HA responded with a decline in photocatalytic process. In the case of 15 mg/L HA, the degradation process reduced more significantly with approximately only 30% of BPA removed after 4 h irradiation. With 10 mg/L HA, the rate of BPA removal was reduced with a degradation rate constant of 0.1473 h^−1^ (Table 2), but not to the extent witnessed with 15 mg/L HA. The negative effect of increasing HA concentration in photocatalytic degradation is reported [61]. This is due to a possible competitive coverage of the surface active sites of the catalyst by the pollutant and HA. In addition, blockage of light penetration due to suspension turbidity by the HA addition can limit the performance. In some cases, induction of indirect alternative photodegradation pathways, and inner filtering capacity of HA that scavenges radicals and possibly act as reactive species precursors cannot be excluded [64,65]. Some mixed behavior is also observed for HA (case of 10 mg/L).

Table 2 depicts degradation process rate of reaction constant (K, h^−1^) and the corresponding correlation coefficient constant (R^2^) for each of the tested photocatalytic experimental parameters.

#### 3.2.6. Evaluation of Visible Light Photocatalytic Activity

**Figure 16:**
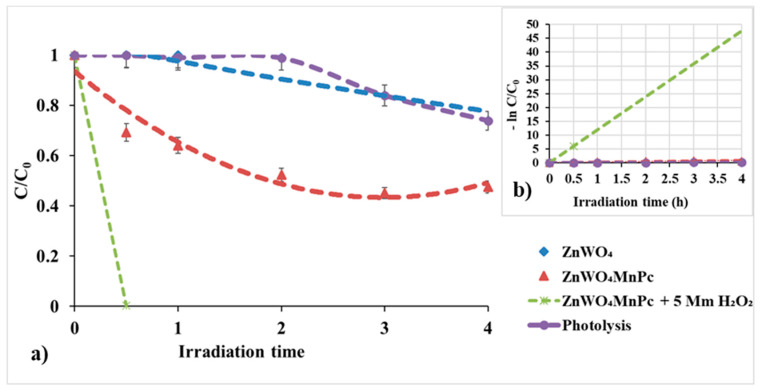
Photocatalytic activity of (**a**) ZnWO_4_, ZnWO_4_MnPc, ZnWO_4_MnPc + 5 mM H_2_O_2_, and photolysis (**b**) respective degradation rate constant of tested materials under 450 nm visible light (1 g catalyst, [BPA]_0_ = 10 mg/L, pH 11).

Employing the same light source used for the near UV-vis range photocatalytic studies of the ZnWO_4_MnPc material described in Section 2.5, extension of its performance under visible light was studied. The maximum wavelength was centered at 450 nm cut off filter of the emission spectrum 300–475 (nm) spectrum. As can be observed from Figure 16, ZnWO_4_MnPc material after 4 h of irradiation removed about 60% of BPA in comparison to ZnWO_4_ material which was only able to remove about 20% BPA. In order to enhance the photocatalytic activity of ZnWO_4_MnPc material, about 5 mM H_2_O_2_ deemed right in concentration to shoulder potential H_2_O_2_ scavenging ability of electron (e^−^) and hydroxyl (HO**·**) radical reactive species [54,60]. ZnWO_4_MnPc material removed almost 100% of BPA after 30 min of irradiation in the presence of 5 Mm H_2_O_2_. This implies that the introduced 5 mM H_2_O_2_ in this concentration, generated more hydroxyl radical (HO**·**) reactive species and possibly could have scavenged generated electrons to prevent charge recombination, hence the degradation reaction rate was highly improved with respect to ZnWO_4_ material without phthalocyanine (Pc) sensitizer [54]. From Table 2, the degradation rate constant of ZnWO_4_MnPc +5 mM H_2_O_2_ is approx. 12 h^−1^ in comparison to 0.2411 h^−1^ for ZnWO_4_MnPc, showing the former performed 50 times better than the later with no added H_2_O_2._ In contrast to photolysis effect towards BPA removal under UV light, just about a little over 20% BPA removed at 4 h irradiation (Figure 16) under the visible light. The non-significant BPA removal under visible light agrees with the almost double value of about 40% obtained under UV where BPA exhibited strong absorption [56]. On the other hand, with the addition of 5 mM H_2_O_2_, an impressive performance was recorded over bare photolysis and catalyst without PC sensitizer and or H_2_O_2_ pointing to its almost non significance under this light condition (Figure 16, Table 2).

Considering the performance of ZnWO_4_MnPc towards BPA degradation in the present study and matching it with previously studied heterogeneous photocatalytic degradation systems for BPA or some other organic pollutants with respect to investigated conditions of irradiation time, wavelength of light source, pH, initial concentration of pollutant, nature of catalyst and dosage, reactor designs, water matrix effects, amongst several other operation parameters; indeed, these array of factors do to a large extent influence organic pollutant photodegradation and removal efficiency. In similar studies like the present one, various researchers have investigated different photocatalysts materials ranging from titanium dioxide to zinc, silver, bismuth, and carbon based for the photodegradation of BPA while employing different light sources, wavelength of irradiation, time of irradiation, initial BPA concentrations, pH, reactor designs achieving diverse array of BPA removal efficiency [66]. On the other hand, investigated TiO_2_ sensitized aluminum phthalocyanine for the degradation of chlorophenol in water under visible irradiation achieved almost 100% degradation after 5 h, while for catechol, it took 6 h to degrade about 40% with the same catalyst [67]. In the same vein, Souza et al.; employed nitrogen doped and copper (ii) phthalocyanine tetracarboxylate sensitized titanate and titania dioxide nanotubes for the degradation of rhodamine B (RhB) dye and observed different behavior of the tested catalysts towards RhB degradation efficiency with and without H_2_O_2_, and in the presence and absence of light after 8 h visible irradiation [68]. Connoting all these information with obtained results in the present study, sheds more light on the dynamism inherent in the behavior of different photocatalyst materials in diverse heterogeneous photocatalytic systems running on various operational parameters; hence the discrepancies in removal efficiency of a particular catalyst towards a particular substrate under different irradiation times of exposure measurements.

### 3.3. Proposed Reaction Mechanism

**Scheme 2:**
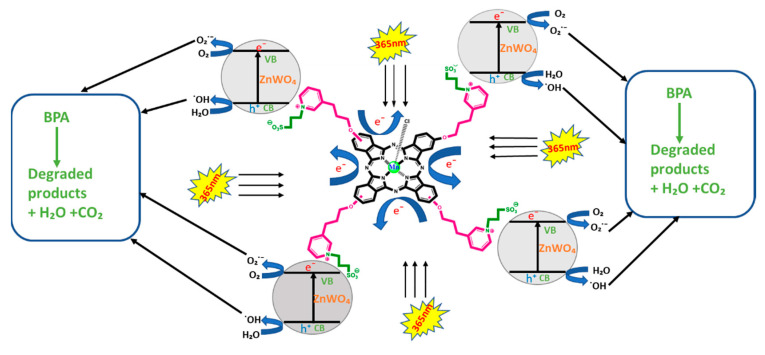
Proposed reaction mechanism for ZnWO_4_MnPc photocatalyst material.

Based on the test for the detection of responsible reactive species assumed to have driven the studied photocatalytic degradation process, surface charge reaction mechanism was proposed. With the assumption, that photocatalytic degradation of organic compounds proceeds via charge carriers like electrons (e^−^), holes (h^+^), and reactive oxygen species like superoxide anions (O_2_^−^), hydroxyl radicals (HO**·**), singlet oxygen (^1^O_2_), the surface reaction mechanism involved in the degradation process of BPA by ZnWO_4_MnPc catalyst was proposed to have followed conventional mechanism with involvement of the most common reactive species [69]. From Figure 13, it was observed that probed reactive species (O_2_^−^, HO**·**, and h^+^) were all involved in the photodegradation process of BPA, hence there were no outright BPA removal inhibition upon the addition of the chemical scavengers except for the hole (h) scavenger probe which after all, did not show so much significant effect from the rest tested two species of superoxide anion (O_2_^−^), and hydroxyl radical (HO**·**). Upon irradiation the ZnWO_4_MnPc catalyst, generates electrons (e^−^) from the Pc molecule which are transferred to the conduction band (CB) energy level as the valence band (VB) holes (h^+^) are simultaneously formed. The hole charge carriers (h^+^) combine with adsorbed surface water molecules to yield hydroxyl radical (HO**·**) while valence band electrons (e^−^) combine with surface O_2_ molecule to yield superoxide anion (O_2_^−^). From the obtained findings of the ROS investigation, the mechanism for ROS generation of the synthesized ZnWO_4_MnPc photocatalyst under UV light is inferred to have proceeded mainly via the generation of hydroxyl radicals (HO**·**) by hole (h^+^) oxidation and accompanied electron (e^−^) reduction and associated superoxide anion (O_2_^−^). These charges are then readily available to attack BPA molecule as it subsequently mineralizes. There may be the possibility of the formation of H_2_O_2_ (Scheme 2). Corroborating at this point, the proposed surface reaction mechanism with other studies already performed with Z-scheme charge transfer mechanisms involving manganese phthalocyanine (MnPc) and other transition metal phthalocyanine complexes for the degradation of organic pollutants, our findings were well supported as hydroxyl radical (HO**·**) reactive oxygen species were also demonstrated to have dominated those oxidation processes [70,71].

## 4. Conclusions

After a successful synthesis and characterization of ZnWO_4_MnPc, the photocatalytic activity of 1 g/L dose of the material under UV 365 nm radiation was evaluated over 10 mg/L of BPA. A 60% degradation at 4 h irradiation time in comparison to the pure ZnWO_4_ which for the same time of irradiation degraded 40% of BPA was achieved. Based on the reactive oxygen species (ROS) probe using scavengers, hole charge carrier (h^+^) was suggested the most responsible for the photooxidation of BPA with a removal mechanism proposed accordingly. Addition of 5 mM H_2_O_2_, on the degradation process caused a significant improvement of BPA removal to above 80% in comparison to about 60% of BPA degraded in the absence of H_2_O_2_ enhancer. Effects of cations and anions were also investigated and 50 mg/L each of Ca^2+^ and K^+^ ions made the biggest impact in slowing down the overall reaction rate during the 4 h irradiation time window. However, 20 mg/L Al^3+^ did not impose a significant inhibitory effect compared to Ca^2+^ and K^+^ ions. Similarly, like in the case of tested Ca^2+^ and K^+^ cations, 20 mg/L NO_3_^−^ and 100 mg/L CO_3_^2−^ ions; suppressed the reaction degradation rate while 200 mg/L Cl^−^ like Al^3+^ showed negligible effect on the overall reaction rate. HA concentrations of 10 mg/L and 15 mg/L affected the reaction rates considerably. The higher content 15 mg/L HA imposed a much significant inhibitory effect than the 10 mg/L HA. In summary, the overall effects of the investigated added ions as well humic acid (HA) made no significant difference amongst themselves in comparison. However, at the concentrations tested and under the experimental conditions applied, they all had a distinct negative effect in the degradation of BPA. With the photocatalytic studies taken over a wide range of pH values, influence of bare photolysis with respect to the ZnWO_4_MnPc catalyst for BPA removal at pH 11 was highlighted and showed considerate and not so significant BPA removal under 365 nm and 450 nm irradiation light sources respectively.

In the near future the full potential of photocatalytic performance of ZnWO_4_MnPc material under visible light conditions will be studied. It has shown activity under 365 nm and potential activity under extended 450 nm visible light irradiation in the presence of 5 mM H_2_O_2_ as an enhancer. The catalyst will be studied towards the improvement of its activity in the visible light range as a standalone phthalocyanine sensitized photocatalyst. The investigation of the charge transfer mechanism more as a novel Z-scheme photocatalyst with some time resolved experiments involving flash photolysis studies will be in focus. Additionally, TiO_2_ (commercial P25) based Pc catalysts will also be studied alongside, and in comparison, while ensuring that pH condition is limited to preferable neutral points.

## Figures and Tables

**Table 1:** Physical properties of synthesized materials ^a^.

Materials	d_XRD_ (nm)	d_TEM_ (nm)	S_BET_ (m^2^/g)	V_p_ (cm^3^/g)	d_p_ (nm)	E_g_ (eV)
ZnWO_4_	13.1	13.4	15.1	0.040	14.0	4.0
ZnWO_4_MnPc	15.1	20.3	11.4	0.035	19.0	4.1

^a^ d_XRD_, crystallite size estimated by XRD; d_TEM_, particle size estimated by TEM_;_ S_BET_, surface area; V_p_, total pore volume and d_p_, average pore size estimated by Barrett, Joyner, and Halenda (BJH) adsorption pore volume and BJH adsorption pore size respectively; E_g_, energy band gap.

**Table 2:** Degradation rate constant (h^−1^) and corresponding correlation coefficient constant (R^2^) of controlled photocatalytic parameters.

Test	Degradation Rate Constant K (h^−1^)	R^2^
Scavenger effects on ZnWO_4_MnPc ^#^		
No Scavenger	0.2661	0.9842
Benzoquinone (BQ)	0.1942	0.9125
Isopropyl alcohol (IPA)	0.182	0.9516
Etheylenediamine tetracetic acid (EDTA)	0.1417	0.9245
H_2_O_2_ effects on ZnWO_4_MnPc ^#^		
0 mM H_2_O_2_	0.2661	0.9842
5 mM H_2_O_2_	0.5442	0.8607
Ion effects on ZnWO_4_MnPc: Anions ^#^		
No ion	0.2661	0.9842
20 mg/L NO_3_^−^	0.1735	0.9815
100 mg/L CO_3_^2−^	0.1823	0.8227
200 mg/L Cl^−^	0.2270	0.9938
Ion effects on ZnWO_4_MnPc: Cations ^#^		
No ion	0.2661	0.9842
20 mg/L Al^3+^	0.2336	0.9674
50 mg/L Ca^2+^	0.1795	0.9006
50 mg/L K^+^	0.1795	0.9006
Humic acid (HA) effects on ZnWO_4_MnPc ^#^		
0 mg/L HA	0.2661	0.9842
10 mg/L HA	0.1473	0.7826
15 mg/L HA	0.0974	0.9219
Photolysis effect/450 nm Visible range test		
ZnWO_4_	0.05830	0.8362
ZnWO_4_MnPc	0.2411	0.9048
ZnWO_4_MnPc + 5 mM H_2_O_2_	11.899	1
UV-365 nm ^#^	0.1595	0.8309
450 nm	0.0586	0.8451

^#^ Tests performed under 365 nm near UV-vis irradiation.

## Supplementary Materials

The following are available online at https://www.mdpi.com/2079-4991/10/11/2139/s1, Figure S1: FT-IR spectrum of BZ-Mn-PS compound, Figure S2: MALD-TOF MS spectrum of BZ-Mn-PS compound, and Figure S3: UV-vis DRS spectra of synthesized materials.
