# Supplementary material for: Synthesis, Characterization, and Photocatalytic Evaluation of Manganese (III) Phthalocyanine Sensitized ZnWO4 (ZnWO4MnPc) for Bisphenol A Degradation under UV Irradiation
Source: Nanomaterials (Basel). 2020 Oct 27;10(11):2139. doi: 10.3390/nano10112139 (PMC7693405; doi:10.3390/nano10112139)
Supplement: Supplementary file 1 [file nanomaterials-10-02139-s001.pdf]

nanomaterials

Article

Synthesis, Characterization, and Photocatalytic Evaluation of Manganese (III) Phthalocyanine Sensitized ZnWO4 (ZnWO4MnPc) for Bisphenol A Degradation under UV Irradiation

Chukwuka Bethel Anucha [1,*], Ilknur Altin [1], Zekeriya Biyiklioglu [1], Emin Bacaksiz [2], Ismail Polat [3] and Vassilis N. Stathopoulos [4]

1 Department of Chemistry, Faculty of Science, Karadeniz Technical University, 61080 Trabzon, Turkey; C.B.Aucha@ktu.edu.tr (C.B.A); ilknurtatlidil@ktu.edu.tr (I.A.); zekeriyab@ktu.edu.tr (Z.B.)

2 Department of Physics, Faculty of Science, Karadeniz Technical University, 61080 Trabzon, Turkey; eminb@ktu.edu.tr (E.B.)

3 Department of Energy Systems, Faculty of Technology, Karadeniz Technical University, 61080 Trabzon, Turkey; ipolat@ktu.edu.tr (I.P.)

4 Laboratory of Chemistry and Materials Technology, General (Core) Department, National and Kapodistrian University of Athens, Psachna Campus, 34400 Evia, Greece; vasta@uoa.gr (V.N.S)

* Correspondence: C.B.Anucha@ktu.edu.tr

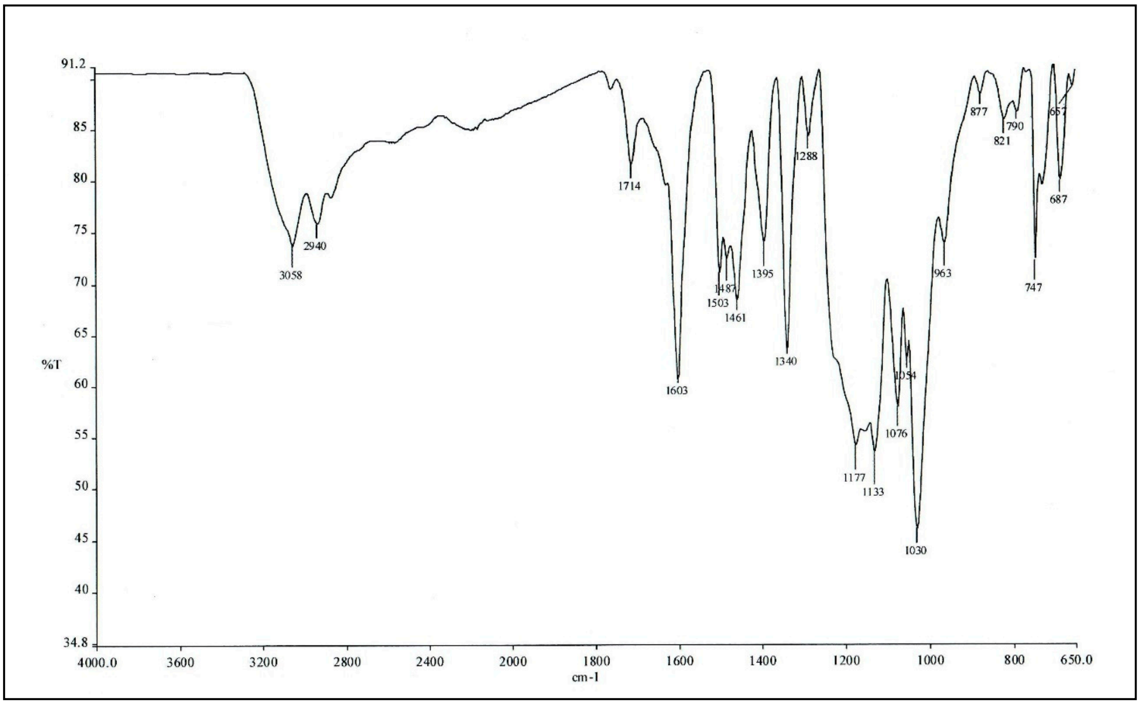

Fig. S1 FT-IR spectrum of BZ-Mn-PS compound

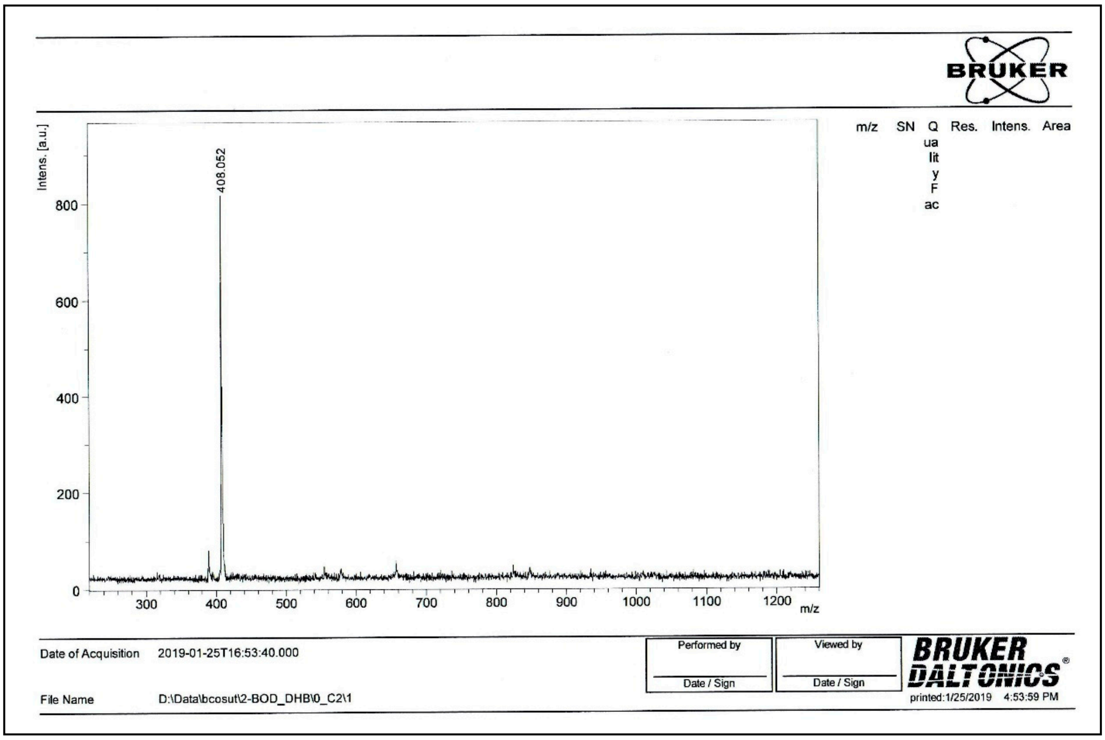

Fig. S2 MALD-TOF MS spectrum of BZ-Mn-PS compound

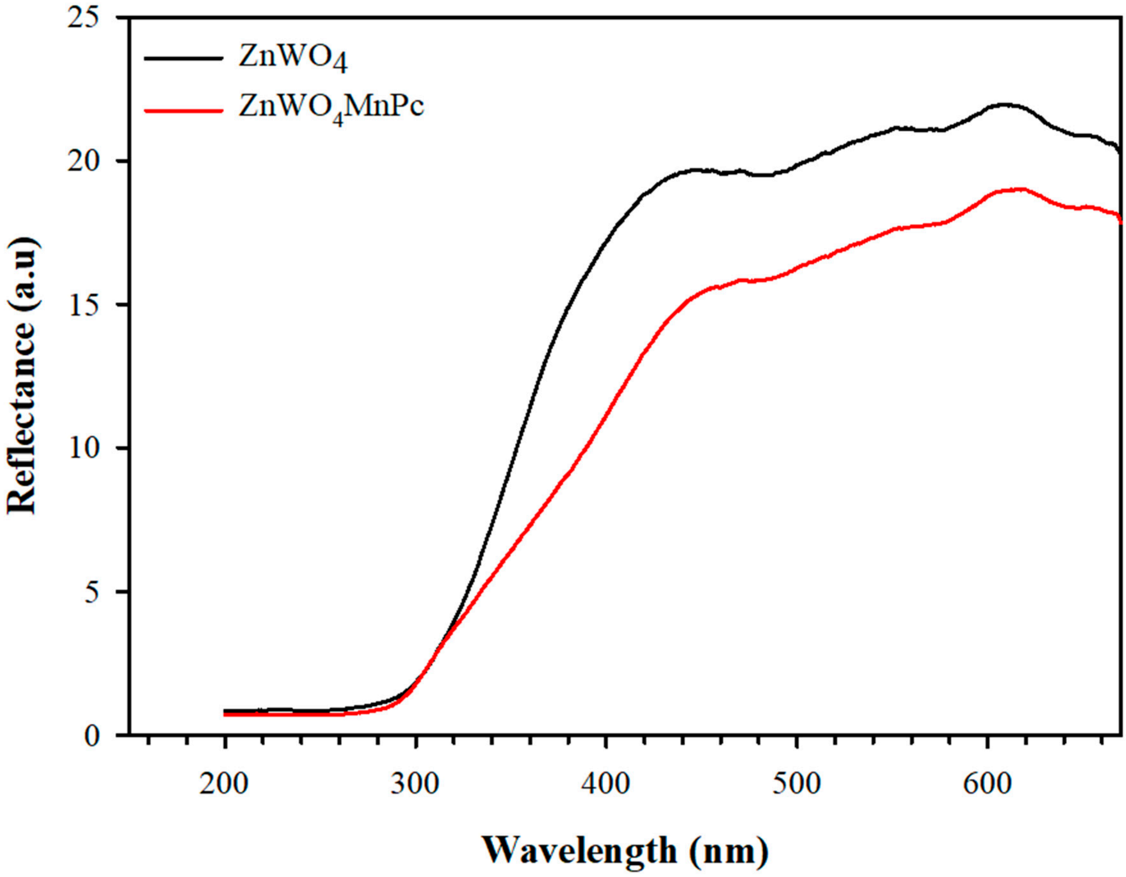

Fig. S3 UV-vis DRS spectra of synthesized materials

© 2020 by the authors. Licensee MDPI, Basel, Switzerland. This article is an open access article distributed under the terms and conditions of the Creative Commons Attribution (CC BY) license (http://creativecommons.org/licenses/by/4.0/).
